# Etiology of severe pneumonia in Ecuadorian children

**DOI:** 10.1371/journal.pone.0171687

**Published:** 2017-02-09

**Authors:** Sivani Jonnalagadda, Oswaldo Rodríguez, Bertha Estrella, Lora L. Sabin, Fernando Sempértegui, Davidson H. Hamer

**Affiliations:** 1 Center for Global Health and Development, Boston University School of Public Health, Boston, Massachusetts, United States of America; 2 Corporación Ecuatoriana de Biotecnología, Quito, Ecuador; 3 Universidad Central del Ecuador, Escuela de Medicina, Quito, Ecuador; 4 Department of Global Health, Boston University School of Public Health, Boston, Massachusetts, United States of America; 5 Section of Infectious Diseases, Department of Medicine, Boston University School of Medicine, Boston, Massachusetts, United States of America; 6 Gerald J. and Dorothy R. Friedman School of Nutrition Science and Policy, Tufts University, Boston, Massachusetts, United States of America; Kliniken der Stadt Köln gGmbH, GERMANY

## Abstract

**Background:**

In Latin America, community-acquired pneumonia remains a major cause of morbidity and mortality among children. Few studies have examined the etiology of pneumonia in Ecuador.

**Methods:**

This observational study was part of a randomized, double blind, placebo-controlled clinical trial conducted among children aged 2–59 months with severe pneumonia in Quito, Ecuador. Nasopharyngeal and blood samples were tested for bacterial and viral etiology by polymerase chain reaction. Risk factors for specific respiratory pathogens were also evaluated.

**Results:**

Among 406 children tested, 159 (39.2%) had respiratory syncytial virus (RSV), 71 (17.5%) had human metapneumovirus (hMPV), and 62 (15.3%) had adenovirus. *Streptococcus pneumoniae* was identified in 37 (9.2%) samples and *Mycoplasma pneumoniae* in three (0.74%) samples. The yearly circulation pattern of RSV (*P* = 0.0003) overlapped with *S*. *pneumoniae*, (*P* = 0.03) with most cases occurring in the rainy season. In multivariable analysis, risk factors for RSV included younger age (adjusted odds ratio [aOR] = 1.9, *P* = 0.01) and being underweight (aOR = 1.8, *P* = 0.04). Maternal education (aOR = 0.82, *P* = 0.003), pulse oximetry (aOR = 0.93, *P* = 0.005), and rales (aOR = 0.25, *P* = 0.007) were associated with influenza A. Younger age (aOR = 3.5, *P* = 0.007) and elevated baseline respiratory rate were associated with HPIV-3 infection (aOR = 0.94, *P* = 0.03).

**Conclusion:**

These results indicate the importance of RSV and influenza, and potentially modifiable risk factors including undernutrition and future use of a RSV vaccine, when an effective vaccine becomes available.

**Trial registration:**

ClinicalTrials.gov NCT 00513929

## Introduction

Pneumonia is the leading infectious cause of mortality among children below 5 years of age, and is responsible for nearly one million deaths annually in this population [1–3]. The World Health Organization (WHO) estimates that the vast majority (over 95%) of the estimated 156 million pneumonia episodes occurring each year among young children takes place in developing countries [4]. Globally, South Asia and sub-Saharan Africa bear the highest burden of pneumonia episodes; fifteen countries within these two regions alone accounted for over two-thirds of pneumonia mortality in 2011 [5–7]. Nevertheless, in Latin America and the Caribbean, community-acquired pneumonia continues to be a prominent cause of high morbidity and mortality among children. Specifically, an estimated 327,000 cases and 12,000–28,000 deaths due to pneumonia occur annually in children in this region [8,9].

Hospital-based studies have contributed to understanding the causes of pneumonia in developing countries [4]. However, determining pneumonia etiology remains challenging due to difficulties in obtaining lower respiratory tract specimens from children [1,10]. The most significant bacterial strains associated with pneumonia in children described in past microbiological studies include *Streptococcus pneumoniae* and *Haemophilus influenzae* type B (Hib), [11–13] followed by *Staphylococcus aureus* and *Mycoplasma pneumoniae* [14]. Furthermore, viral pathogens linked to acute respiratory infections, including pneumonia, include human metapneumovirus (hMPV), adenovirus, influenza types A and B, rhinovirus, respiratory syncytial virus (RSV), and bocavirus [15,16].

Studies examining the etiology of pneumonia in Latin America, in particular Ecuador, are limited. Using blood and lung aspirates from children younger than 5 years from tertiary care hospitals in Bangladesh, Ecuador, India, Mexico, Pakistan, Yemen, and Zambia, bacterial pathogens associated with WHO-defined very severe pneumonia were: *S*. *pneumoniae* and *S*. *aureus*, followed less commonly by *H*. *influenzae*, *Escherichia coli*, and *Pseudomonas aeruginosa* [17]. However, the study conducted by Asghar et al [17] did not assess viral pathogens other than RSV.

As immunization programs have been implemented within developing countries to protect against bacterial pneumonia, viral pathogens have also emerged as prevalent etiologic agents [18]. Specifically, the need for more etiology studies in countries that have introduced both Hib and pneumococcal conjugate vaccines has been recognized in order to define the new distribution of pneumonia etiologies that will help inform future treatment and prevention approaches [12]. In Ecuador, the pneumococcal conjugate vaccine, PCV10, and Hib vaccinations have been introduced, with WHO-UNICEF estimates showing widespread uptake [19].

We recently conducted a trial investigating zinc as an adjunct treatment for severe pneumonia among children in Quito, Ecuador [20] and systematically collected blood and nasopharyngeal samples from participants to determine pneumonia etiology. This manuscript describes the epidemiological characteristics and etiology of pneumonia in this study population.

## Materials and methods

This observational study was performed within the context of a randomized, double blind placebo-controlled clinical trial conducted among children with severe pneumonia, designed to measure the efficacy of daily zinc administration until hospital discharge on the duration of pneumonia symptoms [20]. The original study took place from February 2008 to April 2010 at the Baca Ortiz Hospital in Quito, Ecuador (altitude 2,830 meters) [21]. As the main pediatric referral hospital in Ecuador, Baca Ortiz has approximately 130,000 outpatient and emergency consultations annually, and the most common reason for hospitalization is acute lower respiratory tract infection (ALRI). The Boston University Institutional Review Board and Ethics Committee of the Corporación Ecuatoriana de Biotecnología both approved the study. Written informed consent was obtained from each child’s parent or care provider. The Ethics Committee of the Corporación Ecuatoriana de Biotecnología served as the Data Safety Monitoring Board in order to ensure proper management and protection of study participants.

### Eligibility criteria

Children aged 2–59 months with severe pneumonia were eligible for participation if parental written informed consent was provided. A resident physician entrusted to screen potential study subjects evaluated each child presenting with respiratory symptoms in the emergency room. If the child was considered eligible, a senior study physician conducted a rapid hemoglobin test and complete clinical examination. If the child met the eligibility criteria and was confirmed to have pneumonia, the study physician enrolled the child after obtaining informed consent from the child’s care provider.

Enrollment was based on the presence of cough and/or difficulty breathing, tachypnea (≥50 breaths per minute in children from two to ≤12 months of age; ≥40 breaths per minute in children aged >12 to 59 months), hypoxemia assessed by pulse oximetry (SpO2 <90%) and at least one of the following: rales, wheezing, diminished breath sounds, bronchial breath sounds or pleural rub. Exclusion factors were marasmus or kwashiorkor, measles, pneumonia due to aspiration of a foreign body, hepatic or renal disease, sepsis, congenital abnormalities (cardiac, renal, or genetic), complicated pneumonia (lung abscess, pleural effusion, pneumatocele, atelectasis), very severe pneumonia (using WHO criteria: tachypnea, chest wall indrawing and central cyanosis or inability to drink), severe anemia (hemoglobin below 8 g/dL), and refusal of the primary care provider to provide written informed consent.

At the time of enrollment, study physicians conducted a detailed clinical evaluation consisting of height, weight, respiratory rate, oxygen saturation, axillary temperature, and chest indrawing. Baseline testing also included the following: 1) collection of blood to measure C-reactive protein (CRP), plasma zinc, whole-blood polymerase chain reaction (PCR) for *Streptococcus pneumoniae* and Hib; 2) pulse oximetry; 3) nasopharyngeal aspirate for PCR analysis of atypical bacteria and viruses; and 4) chest X-ray. Study physicians additionally gathered information on demographic characteristics, current illness, and medical history. Further information regarding enrollment and clinical procedures, as well as complete details of follow-up, treatment, randomization, sample size, and clinical procedures are described elsewhere [20].

### Laboratory analyses

At enrollment, blood samples from participants were acquired by venipuncture; hemoglobin was measured using a portable HemoCue Hb 201+ machine (HemoCue, Angelholm, Sweden). As recommended by the WHO, a nasopharyngeal swab was also obtained [22]. Samples were stored at -20°C until processed, in batches during the study. Blood and respiratory samples were transported to the lab following a cold chain protocol.

DNA was extracted from blood samples using the QIAamp DNA blood mini kit (Qiagen, Venlo, Netherlands) according to manufacturer instructions. DNA and RNA were extracted from nasopharyngeal swabs using the HP viral nucleic acid kit (Roche, Basel, Switzerland) according to manufacturer instructions. Extreme DNA/RNA isolation precautions were observed. Leftover clinical samples were stored at -80°C. DNA/RNA quantity and quality were determined by absorbance 260/280 in the spectrophotometer Perkin Elmer and fluorescence by QuantIT (Invitrogen, Waltham, USA). PCR for a housekeeping human gene was used as a succesful extraction control.

### Polymerase Chain Reaction (PCR) of blood samples

Nested PCR for *S*. *pneumoniae* was performed in DNA isolated from blood samples [23]. A preliminary *in vitro* study confirmed that the lower limit of detection was ≤10 colony-forming units/mL [24], while the specificity among control subjects with no identifiable respiratory disorders was 100%. Finnish investigators demonstrated that the same pneumolysin primers had 100% specificity when tested *in vitro* for a variety of pathogens [23]. Genomic DNA ATCC BAA-255D-5 *S*. *pneumoniae* was used as a positive PCR amplification control. PCR for *H*. *influenzae* was performed in DNA isolated from blood using Strain ATCC 33533 *H*. *influenzae* as a control [25].

### Nasopharyngeal (NP) swab PCR

*M*. *pneumoniae* PCR was performed in DNA isolated from NP swabs [26,27], using Genomic DNA ATCC 15531D *M*. *pneumoniae* strain FH as a positive PCR amplification control [28]. Real-time PCR kits, LightMix-40-0303-16, were used for detection of adenovirus, LightMix 40-0222-16 for the detection of *Chlamydophila pneumoniae*, LightMix 40-0225-16 for detection of influenza virus A and B, LightMix 40-0223-16 for detection of parainfluenza virus 1, 2, and 3, LightMix 40-0184-16 for detection of hMPV, and LightMix for detection of RSV were purchased from TibMolBiol (Alelphia, USA). All kits were evaluated and standardized; detection was performed according to manufacturer instructions.

### Data management

The study supervisor checked project-specific case report forms twice weekly for complete, discrepant, inappropriate, and illogical responses. Double data entry was performed with Microsoft Access 2000 (Redmond, WA). SPSS (version 11.5.0 program SPSS Inc., Chicago, IL) was used for validation and cleaning, and SAS 9.3 was used for all analyses.

### Data analysis

Descriptive analysis was conducted for all baseline demographic and clinical characteristics. Frequencies, proportions, means, medians, and interquartile range, as appropriate, were reported. For comparison purposes, etiological characteristics were stratified by the age categories of less than one year and 1–5 years. Chi-squared tests and Pearson correlation coefficients were used to detect differences between proportions and associations among baseline characteristics and etiology. To describe seasonality, both viral and bacterial infections were analyzed by monthly distribution, with parainfluenza types 1, 2, and 3 merged into one category, and influenza types A and B combined. Chi-squared tests were used to detect differences in monthly case distribution for each respective pathogen.

In order to identify potential risk factors, the association of demographic and clinical characteristics with each pathogen was assessed using univariate and multivariable logistic regression; odds ratios (ORs), adjusted odds ratios (aORs), and 95% confidence intervals (CIs) were calculated. Backward selection with entry criteria of α = 0.02 was used to assess variables for inclusion in multivariable logistic regression models. All analyses were conducted using two tail tests at a significance level of α = 0.05.

## Results

**Fig 1:**
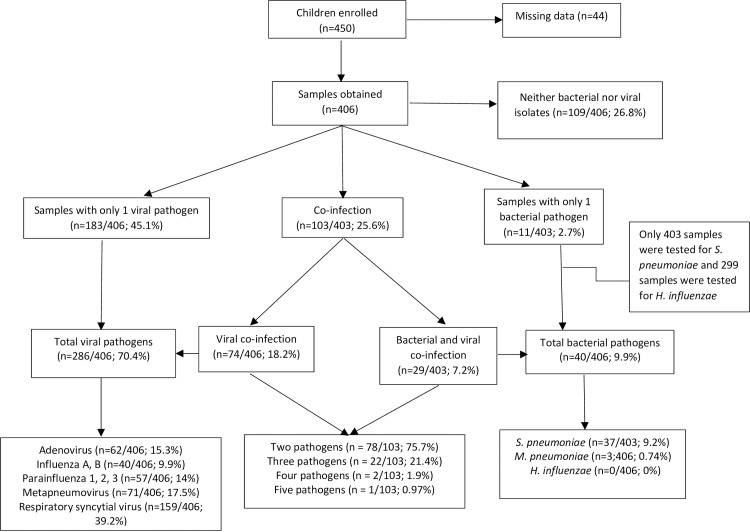
Study profile and analysis of sample pathogens.

Of 450 enrolled children, 406 (90.2%) had blood or nasopharyngeal samples taken, and were subsequently analyzed to determine etiology. Samples were analyzed for bacterial pathogens, viral pathogens, and co-infection with bacterial and viral pathogens (Fig 1). Bacteria or viruses were not detected in 109 (26.8%) of the 406 samples. Only 403 samples were tested for *S*. *pneumoniae;* 299 samples were tested for *H*. *influenzae*. The median age was 9.48 months; 58.6% were less than one year (Table 1). Just over half (54.2%) were male. Among all participants, 190 (46.9%) presented with fever, 77 (19.2%) were underweight, and 70 (18.1%) were stunted.

**Table 1:** Baseline characteristics of children with severe pneumonia (N = 406).

Baseline Characteristics	
**Age**	9.48 ± 12.16 months
< 1 year	238 (58.6%)
1–5 years	168 (41.4%)
**Gender**	
Male	220 (54.2%)
**Household Characteristics**	
Years of maternal education	9 ± 6
Number of people in household	4 ± 2
Number of rooms in household	2 ± 2
Breastfeeding	275 (67.7%)
**Clinical Characteristics**	
Initial temperature^1^ (°C) *	37.6 ± 0.9
Proportion with fever^1^ **	190 (46.9%)
Weight-for-age z (WAZ) score ^2^	-1.0 ± 1.5
Height-for-age z (HAZ) score ^3^	-0.8 ± 1.7
Underweight (WAZ ≤ -2) ^2^	77 (19.2%)
Stunting (HAZ ≤ -2)^4^	70 (18.1%)
Initial respiratory rate (breaths/min)	58.0 ± 9
Respiratory signs: ^1^	
• Pulse oximetry (% SO_2_) • Rales • Bronchial breath sounds • Pleural rub • Whistle	• 80 ± 6.0 • 363 (89.6%) • 48 (11.8%) • 5 (1.2%) • 66 (16.3%)
Before presentation to hospital:	
• Vaccinated^5^ • Provided with antibiotic^6^ • Provided with bronchodilator^6^	• 305 (75.9%) • 27 (56.3%) • 40 (34.7%)
Chest X-ray findings: ^7^	
• Infiltrate • Consolidation • Air trapping • Other***	• 255 (65.9%) • 14 (3.6%) • 54 (14.0%) • 64 (16.5%)

All values are n (%) and median ± IQR. Variability in sample size of characteristics due to missing responses:

^1^n = 405

^2^n = 401

^3^n = 394

^4^n = 386

^5^n = 402

^6^n = 403

^7^n = 387

*Mean and standard deviation.

**Fever (temperature ≥ 37.5°C).

***Other includes pneumothorax and necrosis.

### Viral etiology

Of 406 participants screened, the most common viral isolate was RSV (n = 159; 39.2%), followed by hMPV (n = 71; 17.5%), adenovirus (n = 62; 15.3%), and parainfluenza virus (n = 57; 14.0%) (Table 2). Among 40 (9.9%) children who tested positive for influenza, 29 had influenza type A and 11 had influenza type B. Among children with human parainfluenza, 15 tested positive for type 1, 7 for type 2, and 35 for type 3.

**Table 2:** Pneumonia etiology by age category.

	Age Group N (%)		Total (N = 406)*
Viral Pathogens	< 1 year (n = 238)	1 to 5 years (n = 168)	
Adenovirus	35 (14.7%)	27 (16.1%)	62 (15.3%)
Influenza			40 (9.9%)
Type A	16 (6.7%)	13 (7.7%)	29 (7.1%)
Type B	7 (2.9%)	4 (2.4%)	11 (2.7%)
Parainfluenza			57 (14.0%)
Type 1	10 (4.2%)	5 (3.0%)	15 (3.7%)
Type 2	3 (1.3%)	4 (2.4%)	7 (1.7%)
Type 3	27 (11.3%)	8 (4.8%)	35 (8.6%)
Human metapneumovirus	40 (16.8%)	31 (18.5%)	71 (17.5%)
Respiratory syncytial virus	105 (44.1%)	54 (32.1%)	159 (39.2%)
**Bacterial Pathogens**			
*Streptococcus pneumoniae* (n = 403)	20 (8.5%)	17 (10.1%)	37 (9.2%)
*Mycoplasma pneumoniae*	0 (0%)	3 (1.8%)	3 (0.74%)
**Haemophilus influenzae (n = 299)	175 (58.5%)	124 (41.5%)	0 (0%)

*There were 406 total nasopharyngeal samples for respiratory viruses and *M*. *pneumoniae*, 403 for *S*. *pneumoniae* and 299 for *H*. *influenzae*.

**Since all 299 *H*. *influenzae* samples were found to be negative, the frequencies for age category represent those children that had samples taken.

Significant differences across age category among viral pathogens were found for RSV and human parainfluenza type 3 (HPIV-3) (Table 2). Of the 159 children who tested positive for RSV, 105 (66.0%) were less than one year old. These children had 1.67 times the odds of RSV infection compared to children aged 1–5 years (95% CI, 1.10, 2.52; *P* = 0.015). Of the 35 children who tested positive for HPIV-3, 27 (77.1%) were aged less than one year. These younger children had 2.56 (95% CI, 1.13, 5.78; *P* = 0.02) times the odds of HPIV-3 relative to children aged 1–5 years. The odds of infection did not differ by sex for any viral pathogen.

### Bacterial etiology

*S*. *pneumoniae* was the most common bacterial pathogen isolated (9.2%; 37/403) followed by *M*. *pneumoniae* (0.74%; 3/406). Testing of the bacterial pathogen *H*. *influenzae* was discontinued after finding zero positive samples among 299 tested. There were no significant differences across age category for either bacterial pathogen.

### Co-infection by both bacterial and viral pathogens

Co-infection with more than one pathogen was present in 25.6% (103/403) of children. Of these 103 participants, 78 (75.7%) displayed co-infection with two pathogens, 22 (21.4%) with three pathogens, two (1.9%) with 4 pathogens, and one (0.97%) with 5 pathogens. The most common dual infection combinations were RSV and hMPV in 20 (19.4%), adenovirus and RSV in 14 (13.6%), *S*. *pneumoniae* and RSV in 9 (8.7%), HPIV-3 and RSV in 7 (6.8%), influenza A and adenovirus in 3 (2.9%), and *S*. *pneumoniae* and adenovirus in 3 (2.9%). The most prevalent triple infection combinations were the following: hMPV, adenovirus and RSV in 5 (4.9%); *S*. *pneumoniae*, hMPV and RSV in 3 (2.9%); hMPV, HPIV-3 and RSV in 3 (2.9%); and hMPV, influenza A and RSV in 2 (1.9%). Two participants were infected with 4 pathogens: RSV, hMPV, *S*. *pneumoniae*, and HPIV-2 were isolated in one individual; hMPV, HPIV-2, HPIV-3, and RSV were isolated in the other. One participant was found to have bacterial and viral co-infection with 5 pathogens. The pathogens isolated from this individual included *S*. *pneumoniae*, hMPV, adenovirus, HPIV-3, and RSV. Multiple infections with *S*. *pneumoniae* in combination with RSV, hMPV, adenovirus, influenza A and B, HPIV-2 and HPIV-3 were detected in 27/403 (6.7%) samples. Of these 27 multiple co-infections, *S*. *pneumoniae* was most commonly present with RSV in 14 samples, followed by hMPV in 9 samples, and adenovirus in 6 samples. Dual infection with *M*. *pneumoniae* was present in 2 samples, containing RSV and adenovirus.

Significant associations were present between infection by RSV and hMPV, RSV and influenza A, as well as RSV and HPIV-1. Specifically, individuals infected with RSV had 1.90 (95% CI, 1.13, 3.18; *P* = 0.014) times the odds of hMPV infection compared to individuals without RSV. In contrast, individuals with influenza A had reduced odds of having RSV (0.23, 95% CI, 0.078, 0.67; *P* = 0.007) compared to individuals without influenza A. Furthermore, individuals with HPIV-1 had reduced odds of having RSV (0.11, 95% CI, 0.014, .81; *P* = 0.03) compared to those without HPIV-1 infection.

### Seasonality

**Fig 2:**
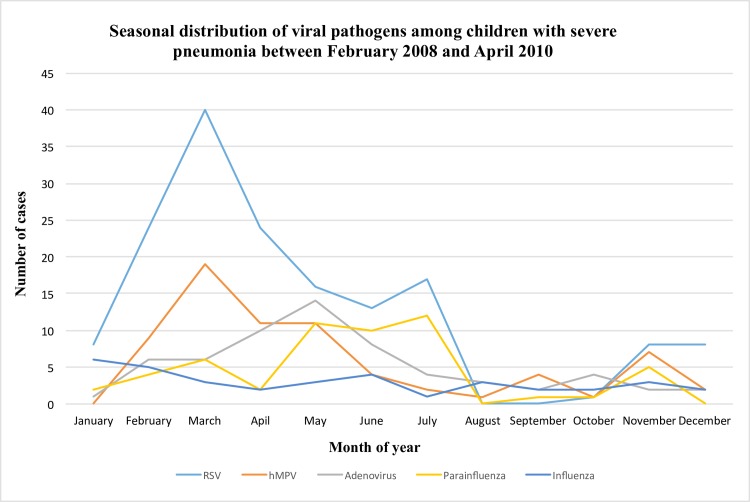
Seasonal distribution of viral pathogens among children with severe pneumonia between February 2008 and April 2010.

**Fig 3:**
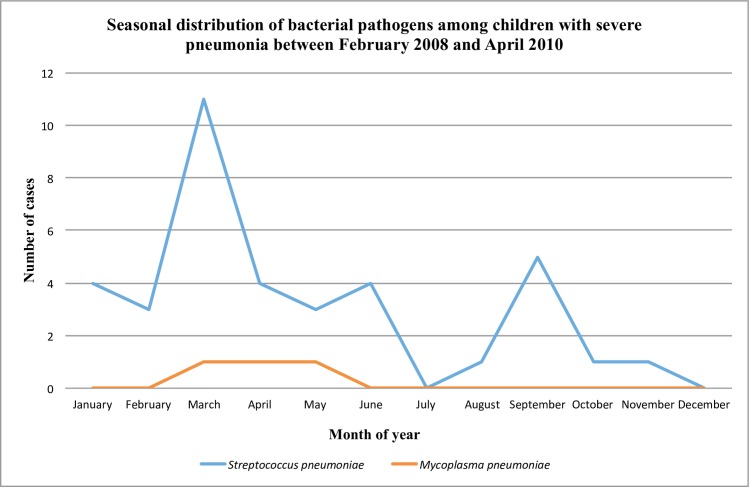
Seasonal distribution of bacterial pathogens among children with severe pneumonia between February 2008 and April 2010.

The climate of Quito consists of a dry (June to September) and rainy season (October to May). Figs 2 and 3 exhibit the number of cases of viral and bacterial pathogens during specific months of the year. The distribution of viral pathogens displays an increasing trend from October to May. Specifically, the circulation of viral pathogens was greatest between January and July, and lowest from July to August. Among all pathogens, RSV (*P* < 0.0001), parainfluenza (*P* < 0.0001), hMPV (*P* = 0.01), and *S*. *pneumoniae* (*P* = 0.03) significantly differed across month of the year. RSV circulation was greatest between February and April, and moderate between May and July with a slight peak in November (Fig 2). A similar circulation pattern was displayed by hMPV, with the highest number of cases occurring between February and May, and another peak in November. Parainfluenza cases were highest between May and July. The circulation of *S*. *pneumoniae* was greatest between February and March, with moderately high circulation from July to September (Fig 3). Circulation of *M*. *pneumoniae* was greatest between February and June, and was negligible during other months.

### Demographic and clinical risk factors associated with specific pneumonia etiologies

Using backward selection, significant associations between baseline demographic and clinical characteristics were found for hMPV, RSV, HPIV-3, and influenza A. In univariate analysis, decreased odds of influenza A infection was associated with maternal education (OR = 0.85, 95% CI, 0.76, 0.96; *P* = 0.001), pulse oximetry (OR = 0.93, 95% CI, 0.89, 0.97; *P* = 0.002), and presence of rales (OR = 0.32, 95% CI, 0.13, 0.81; *P* = 0.02). For hMPV, participants treated with a bronchodilator at baseline had 2.5 higher odds of infection compared to those without a bronchodilator (95% CI, 1.5, 4.2; *P* = 0.0006). An increased odds of infection due to younger age was associated with RSV (OR = 1.7, 95% CI, 1.1, 2.5; *P* = 0.02) and HPIV-3 (OR = 2.6, 95% CI, 1.1, 5.8; *P* = 0.02).

Table 3 describes the multivariable analysis of demographic and clinical factors associated with RSV, HPIV-3, influenza A, and hMPV. The following variables were included in the multivariable logistic regression models: younger age, maternal education, presence of bronchodilator, fever, initial respiratory rate, underweight, pulse oximetry, and presence of rales. Participants who were underweight (aOR = 1.8, 95% CI, 1.0, 3.1; *P* = 0.04) and aged less than one year (aOR = 1.9, 95% CI, 1.2, 3.0; *P* = 0.01) had increased risk of RSV infection. Decreased odds of influenza A infection was associated with increase in years of maternal education (aOR = 0.82, 95% CI, 0.72, 0.94; *P* = 0.003), increase in oxygen saturation (aOR = 0.93, 95% CI, 0.89, 0.98; *P* = 0.005), and presence of rales (aOR = 0.25, 95% CI, 0.09, 0.68; *P* = 0.007). Lastly, for HPIV-3, elevated baseline respiratory rate was associated with reduced odds of infection (aOR = 0.94, 95% CI, 0.88, 0.99; *P* = 0.03) whereas younger age was associated with increased risk (aOR = 3.5, 95% CI, 1.4, 8.6; *P* = 0.007).

**Table 3:** Multivariable regression analysis of common pathogens by explanatory variables.

	RSV (n = 159)		HPIV-3 (n = 35)		Influenza A (n = 29)		hMPV (n = 71)	
Characteristic	Adjusted Odds Ratio (aOR) (CI)	*P* Value	aOR (CI)	*P* value	aOR (CI)	*P* value	aOR (CI)	*P* value
Younger age	1.9 (1.2, 3.0)	0.01*	3.5 (1.4, 8.6)	0.007*	0.79 (0.36, 2.1)	0.58	1.0 (0.57, 1.9)	0.92
Maternal education	1.0 (0.96, 1.1)	0.58	1.1 (0.97, 1.2)	0.20	0.82 (0.72, 0.94)	0.003*	1.0 (0.93, 1.1)	0.87
Presence of bronchodilator	1.1 (0.7, 1.7)	0.70	0.77 (0.35, 1.7)	0.51	0.55 (0.22, 1.4)	0.22	3.0 (1.7, 5.1)	.0001*
Fever	1.2 (0.76, 1.8)	0.46	0.89 (0.42, 1.9)	0.77	1.1 (0.49, 2.6)	0.79	1.5 (0.87, 2.7)	0.14
Initial respiratory rate	1.02 (0.99, 1.1)	0.14	0.94 (0.88, 0.99)	0.03*	1.0 (0.97, 1.1)	0.43	0.99 (0.96, 1.03)	0.82
Underweight	1.8 (1.0, 3.1)	0.04*	1.5 (0.60, 3.9)	0.38	1.8 (0.71, 4.4)	0.22	0.98 (0.47, 2.1)	0.97
Pulse oximetry	0.99 (0.96, 1.0)	0.71	1.0 (0.97, 1.1)	0.43	0.93 (0.89, 0.98)	0.005*	1.0 (0.98, 1.07)	0.37
Rales	1.4 (0.67, 2.7)	0.39	2.0 (0.46, 9.0)	0.35	0.25 (0.09, 0.68)	0.007*	1.7 (0.63, 4.5)	0.31

Values that were significantly different (p<0.05) are indicated by the symbol *. The number of infections by RSV, HPIV-3, influenza A, and hMPV are smaller than those presented in Table 2 because analysis was restricted to those for whom all baseline measurements were available.

## Discussion

There is a pressing need to better understand the etiology of pneumonia in children, especially in resource-limited countries, in order to develop vaccines for prevention and more effective treatments. This study is the first to describe the major viral and bacterial pathogens associated with severe pneumonia among children living in the Andean region of Ecuador. The most common viral pathogens were RSV, hMPV, adenovirus, and parainfluenza, findings consistent with results from a household cohort study conducted in Peru [29,30]. Like the Peruvian study [29], we found that RSV and hMPV were responsible for a substantial portion of lower respiratory infection etiology among young children, and co-infection by RSV and hMPV was common. The proportion of RSV (39.2%), hMPV (17.5%), and RSV-hMPV co-infection (4.9%) detected in our entire sample was larger than a recent study conducted in Guatemala describing hMPV in 9%, RSV in 24%, and RSV-hMPV co-infection in 0.7% of ARI cases [31]. Consistent with past research, younger age was a risk factor for HPIV-3 as well as RSV [32–34]. HPIV serotypes 1, 2, and 3 are common etiologic agents of respiratory illness, but HPIV-3 frequently is associated with severe pneumonia in young infants [35,36]. Since RSV and HPIV-3 infect children in the first few months of life, our findings highlight the need for a vaccine against RSV and HPIV-3 for use in early infancy.

In Ecuador, October-May comprises the rainy or winter season, and June-September the dry or summer season. Studies conducted in Colombia and Brazil describe the climactic impact of rainfall on viral pneumonia circulation, and showed that the circulation of RSV and hMPV was greatest during the late winter months that encompass the rainy season [37,38]. In our study, RSV and hMPV showed similar seasonality patterns with more cases in February-May and moderate peaks between June and July. Our findings of seasonality are further substantiated by epidemiological monitoring conducted by PAHO, which describes peak RSV activity within the months of March-May specifically in the Andean region [39].

Furthermore, the seasonality observed in this work is similar to studies of bacteremic pneumococcal pneumonia coinciding with viral pneumonia, which described a greater number of cases in late winter [40,41]. Specifically, circulation of *S*. *pneumoniae* was highest during February-March with moderate peaks between July and September, which overlapped with RSV and hMPV cases. Weinberger et al [41] described the parallel pattern of RSV and *S*. *pneumoniae* as providing new information that may help to understand and predict the potential impact of new vaccines.

Current evidence implicates multiple pathogen infection involving viral and bacterial-viral etiologic agents with severe pneumonia [18]. In our study, 18.0% of children had triple viral infection, which is in agreement with a recent review that found dual or triple viral infection detected among 10–20% of children with pneumonia [42]. Interestingly, co-infection by *S*. *pneumoniae* was present with all viral pathogens with the exception of HPIV-1 (although there were relatively few HPIV-1 isolates), in combination with two, three, and four other viral pathogens. The most frequent combination that we detected was *S*. *pneumoniae* with RSV. Similar circulation patterns and co-infection between these two pathogens inspires further study into the interaction between these two causative agents, which has been under investigation *in vivo* and *in vitro* [43,44]. Overall, the interaction between bacterial and viral pathogens in terms of temporality and severity of childhood pneumonia is unclear and merits further investigation [45]. Ultimately, understanding these infection patterns among communities in developing countries is vital to implementing effective strategies to prevent pneumococcal pneumonia [46,47].

In Ecuador, the Hib and pneumococcal conjugate (PCV) vaccines were introduced in 2003 and 2010, respectively [19]. Specifically, PCV7 was introduced in the Ecuador national immunization program in 2010, which was then replaced by PCV10 in 2011 [48–50]. PCV has been recommended for infants at 2, 4, and 6 months, and recent WHO-UNICEF report estimates describe an increase in vaccine uptake from 2% in 2009 to 81% in 2015 [19]. The absence of Hib in our sample was likely due to use of the Hib vaccine. Hib vaccine uptake has been excellent; a recent WHO report indicated that vaccine coverage increased from roughly 54% in 2003 to 99% in 2011, and to 87% in 2014 among children aged 12–23 months [19]. We discontinued testing for Hib after the first 299 children, since no samples tested positive for the pathogen, a finding suggesting that this vaccine has had a major impact on Hib infection. The limited detection of *M*. *pneumoniae* might have been due to the younger age of our study participants. Recent studies have shown school-aged children to typically have a higher risk of infection with *M*. *pneumoniae* in comparison to younger children and infants [51,52]. Recent reports have described a favorable reduction of community-acquired pneumonia and invasive pneumococcal disease following PCV uptake among developing countries [18,53,54]. Similarly, the development of an RSV vaccine is underway, tailored to target populations such as infants, children, and pregnant mothers [55–57]. Hospitalization and morbidity due to RSV among children in developing countries [58,59] strengthens the need for RSV vaccine implementation in Ecuador, and by extension the Andean region.

In Ecuador, the seasonal influenza vaccination was incorporated in the national immunization schedule in 2006 [60], and estimates show 46% coverage among children 6 months-4 years as of 2015 [61,62]. The presence of influenza-associated severe pneumonia in developing countries is important because influenza is potentially vaccine-preventable, and can predispose to subsequent infection with other pathogens [15,63]. A study conducted in El Salvador [64] described influenza virus among 6% of 608 cases of severe pneumonia, comparable to 9.9% influenza and 3.4% influenza co-infection cases detected in our study. Given the presence of this pathogen in Latin America, we believe that Ecuadorian children would benefit from increased coverage of the influenza vaccine.

Recent studies have shown that severe pneumonia is more prevalent among high altitude regions [65,66], and describe the impact of high altitude on hypoxemia and elevated respiratory rate [67–69]. In addition to age, undernutrition, lack of breastfeeding, and lack of maternal education have also been described as pneumonia risk factors [70–72]. Considering the elevation of Ecuador, we aimed to explore possible differences in clinical presentation and socio-demographic factors among the prominent pathogens detected in our sample. Specifically, associations between baseline clinical factors and viral pathogens were found for RSV, HPIV-3, and influenza A. Our results indicated that an increased respiratory rate at the time of hospital admission was associated with decreased odds of HPIV-3. In contrast, we also found that increase in pulse oximetry percentage was associated with decreased odds of influenza A. Previous studies conducted in Colombia and Peru showed that low oximetry was a better indicator of pneumonia than fast breathing, and that Andean Peruvian children are at higher risk for hypoxia with ARI because of the high altitude [30,73]. Howard et al [74] similarly described the impact of high altitude on lower baseline oxygen saturation, impaired respiratory ciliary function, pulmonary vasoconstriction, and susceptibility to respiratory illness among children in Peru. Thus, we hypothesized that the associations we found in Ecuador between viral pneumonia and pulse oximetry may result from high altitude, among many factors contributing to hypoxemia.

Lastly, the associations found between being underweight and increased odds of RSV, and between years of maternal education and decreased odds of influenza A support the role of socio-demographic characteristics as risk factors for pneumonia. In our study, underweight (weight-for-age z score <2 standard deviations) was considered a marker of undernutrition in line with current literature [72,75,76]. A meta-analysis conducted by Jackson et al [75] described 6 studies in developing countries that reported an association between being underweight and risk of severe ARI, while recent studies have described low birth weight as a risk factor for severe RSV infection among children [77–79]. However, the association between being underweight and RSV infection, particularly among cases of severe pneumonia, remains unclear. Similarly, there are few studies directly exploring an association between maternal education and influenza A among cases of severe pneumonia. Nonetheless, maternal education as a risk factor for severe, community-acquired pneumonia has been described in developing countries [70,80,81].

At the time our study was planned and implemented, the role of human rhinovirus/enterovirus (HRV/ENT) and human bocavirus (hBoV) as etiological agents for pneumonia, particularly severe pneumonia, was uncertain. Traditionally, HRV/ENT has been associated with upper respiratory tract infections, especially the common cold [82–84]. However, recent literature has established these pathogens as causative organisms for lower respiratory tract infection and community-acquired pneumonia [83,85,86]. In addition, the detection of HRV/ENT among asymptomatic individuals with acute lower respiratory infection has complicated interpretation of the role of these viruses in respiratory infections [74,87]. Furthermore, although hBoV is relatively a newly described pathogen as a cause of community-acquired pneumonia [83,85,88], its clinical significance and etiological role requires further clarification [42,87,89,90]. Since neither HRV/ENT nor hBoV were well described as causes of childhood pneumonia when our study was designed, these pathogens were not included in the PCR testing we decided to use for determining severe pneumonia etiology.

Strengths of our study included the standardized definition of pneumonia, close monitoring of respiratory signs, training of the study team, and standardization of laboratory measurement techniques [20]. Limitations were a lack of community-matched controls and routine bacteriologic test procedures (e.g., blood cultures, induced-sputum Gram stain and culture). The original study exhibited a higher early dropout rate than anticipated, but this was addressed by increasing the sample size [20]. Furthermore, although not widely implemented, PCR was used to test for *S*. *pneumoniae*, which has recently shown high sensitivity in the detection of pneumococcal pneumonia [91,92]. In this study, however, we could not serotype *S*. *pneumoniae* isolates. In addition, the use of nasopharyngeal samples to detect viral pathogens provides only indirect evidence of pneumonia etiology. This method was utilized because obtaining pulmonary samples from lower airways and blood samples from children would have been logistically challenging and overly invasive. Finally, a limited number of cases for some pathogens constrained interpretation of seasonal trends.

In conclusion, our study describes the bacterial and viral etiology as well as manifestations of co-infection attributable to pediatric pneumonia in Ecuador. Notably, results from the present study illustrate the need for an RSV vaccine, and suggest increased coverage of the influenza vaccine for children in Ecuador as well. Our findings of overlapping circulation patterns of RSV and bacteremic pneumonia, particularly during the rainy season, provide information that may help to understand and predict the impact of vaccines for RSV (future) and *S*. *pneumoniae* in this region. Furthermore, the isolation of demographic and clinical risk factors for viral pathogens emphasizes the need for additional research in this area specific to climate and altitude in the Andean region. This work provides an important foundation for further studies in this region that are critically needed to develop improved strategies for the treatment and prevention of pneumonia among Latin American children.

## Supporting information

S1 File Database. (XLSX)

S2 File Variable Description Chart. (XLSX)
